# Formulation of a corporate governance index for banking sector: The GIB.X62

**DOI:** 10.1016/j.heliyon.2023.e15253

**Published:** 2023-04-08

**Authors:** Zouhour El-Abiad, Udo Braendle, Hani El-Chaarani

**Affiliations:** a Beirut Arab University, Lebanon; b ESA Business School, Beirut; c IMC University of Applied Sciences Krems, Austria

**Keywords:** Bank, Corporate governance, Index, Internal governance mechanisms

## Abstract

The purpose of this research is to define a new international corporate governance index for the banking sector (GIB.X62) based on 62 criteria and 7 internal performance indicators related to board of directors, internal audit, compensation, risk management, nomination, compliance, ethics, transparency and disclosure. The new index model was applied on 7 different banks from US, France, Spain, Italy, Lebanon, Egypt and Jordan in 2021. The GIBX(62) can be generalized and applied by the international banks to measure their corporate governance efficiency. In addition, the GIBX(62) can be used by shareholders, depositors and regulators at the national and international level to monitor the process of corporate governance practices in banking sector.

## 1 Introduction

The analysis of corporate governance mechanisms and its relationship with the performance of banking sector is not a recent topic in financial field. The international financial crisis of 2008 has shown that the weakness and deficiencies of corporate governance mechanisms were the major causes behind the failure and the bankruptcy of many large banks. Despite the injection of billions of dollars and euros by US Federal Reserve and European Commission, many banks such as Lehman Brothers and Washington Mutual Bank went bankrupt.

Many international professional boards and scholars have argued that the corporate governance of banking sector should be reinforced and reviewed. BASEL committee have published in 2015 over 15 recommendations to improve the monitoring system in banking sector [1]. The International Monetary Funds [2] and the BASEL committee [3] have explored the causes of the international financial crisis of 2008. They have indicated that the corporate governance deficiency was a major cause of the failure of banking industry and have recommended banks to improve their corporate governance mechanisms. The board members of European commission [4] have published a green paper in 2010 in which they have recommended European banks to reinforce their corporate governance mechanisms. The OECD [5] have published a set of corporate governance recommendations. The purpose is the protection of interests of the various stakeholders.

The importance of corporate governance mechanisms in increasing the financial performance of firms was revealed by many theories. For example, the agency theory [6] has recommended the owners to implement different controlling tools to monitor the director and eliminate any expropriation behavior within any firm. The entrenchment theory [7] has proposed the adoption of efficient corporate governance mechanisms to reduce the entrenchment behavior by the CEO.

Looking at the corporate governance literature, the need to improve the systems has been widely discussed to reduce risks in the financial services sector. Studies have shown that performance of banks has been correlating with efficient corporate governance processes [[8], [9], [10], [11]]. To address the agency problems in the sector several authors have recommended that banks should work towards improving governance structures [[11], [12], [13], [14]]. They also encouraged governments to review their financial laws to prevent the manipulation of corporate governance mechanisms.

Despite the huge number of theories, research papers and international recommendations related to corporate governance mechanisms in the financial services industry, the literature review does not present a clear definition of efficient mechanisms that could be implemented in the banking sector. Some scholars have focused on the ownership structure while others have considered other dimensions such as the existence of professional committees and the hiring of independent members on the board of directors.

The aim of this paper is to fill the literature gap by proposing a new index in the area of international corporate governance specifically to be applied by banks. The role of this index is to provide bankers a tool to monitor the efficiency of their corporate governance designs. This is quite different to other approaches relying on public legal protection for example [14]. Whereas such indices rely on factors such as transparency, accountability, and risk management, our index focuses on the efficiency of the corporate governance designs. The practicability is one of the key factors.

The second objective of this paper is to explore the proposed index within different banks from several countries to reveal its capacity to be implemented by bankers worldwide. The outputs of this exploration could be very useful by bankers and financial regulators since they provide a practical example of the proposed corporate governance index.

The remainder of this contribution is presented in the this format: in section one the component of internal indicators in the banking industry are identified. The second section defines the Index for the banking sector in corporate governance (CGIB). Section three presents the implementation of the proposed index in different banks from several countries. Section four discusses the implications and finishes.

## 2 Definition of the internal corporate governance indicators in banking sector

### 2.1 Importance of corporate governance index for banking sector

Numerous studies and theories have encouraged the implementation of efficient corporate governance mechanisms to monitor the agent (CEO) of firms and enhance their financial performance. Jensen and Meckling [6] recommended the implementation of internal corporate governance tools to eliminate the expropriation of private benefits by the agent when there is a separation between ownership and management. They proposed several external and internal corporate governance tools related to compensation plans, dividend payout strategy, ownership structure and independent members on board of directors to minimize the traditional agency conflicts between manager and owners.

In the same line, entrenchment theory [7] found that the internal corporate governance tools related to the existence of independent members on board of directors and dividend payout policy could be used by owners to limit the expropriation of private benefits by the agent.

In case of banks, Vicente-Ramos et al. [15] and El-Chaarani and El-Abiad [14], argued that the existence of efficient corporate governance is an important tool to reduce the agency conflicts that could occur between debtholders, managers, and owners. They stated that managers and manager-owners can select risky investments and strategies to raise their private benefits without considering the interests of external owners and debtholders. They also argued that managers can abuse their authority within banks to expropriate the excess of free cash-flows derived from saving accounts [16].

The international financial regulators like OECD, European Commission and BASEL, also recommended to the financial services industry to enhance their systems to minimize banks risks and protect owners and depositors from any manipulation by the managers [[2], [3], [4], [5]].

The literature review and the analytical screening of a corporate governance index within the banking sector showed that there are seven corporate governance tools with the potential to be employed by banks, namely, 1-board of directors, 2-risk management, 3-internal audit, 4-remuneration plans, 5-compliance and ethics, 6-nomination, and 7-disclosure and transparency.

### 2.2 Board of directors' indicator (BDI)

Based on agency theory [6], the main objective of board of directors is to monitor the management of any firm and safeguard the interests of stakeholders (owners and debtholders). In case on banks, the board of directors are responsible for strategy development, defining appropriate structures, and controlling the operational and financial decisions of executives. The OECD [5] and BASEL committee [2,3] have recommended banks to hire and organize their board of directors to control their risks and optimize their financial decisions. They provide bankers more than 20 recommendations related to board of directors. Numerous scholars in banking sector [17,18] proposed other recommendations related to the diversity, size, and experience of board. Love et al. [19], and Fernandes et al. [12] have revealed that several criteria related to board independency and absence of duality can improve the financial performance of banks. other studies. Based on the literature review, the criteria presented in Table 1 should be applied.

**Table 1:** Board of directors' indicator (BDI).

Symbol	Criterion	Value of each criterion
BDI(1)	Size of board	This criterion equals to: 5 if: 9 < board size <12, 4 if: 8 < board size <13, 3 if: 7 < board size <14, 2 if: 6 < board size <15, 1 if: 5 < board size <16, 0 otherwise
BDI(2)	Number of Independent	This criterion equals to: 5 if the level of independent members:> 50% 4 if: 40% < % independent members <50%, 3 if: 30% < % independent members <40%, 2 if: 20% < % independent members <30%, 1 if: 10% < % independent members <20%, 0 otherwise
BDI(3)	Duality between chairman of board and CEO	This criterion equals to 5 in case of separation between chairman and CEO 0 otherwise
BDI(4)	Meetings number	This criterion equals to: 5 if the meeting number is 12 and above 4 if: meetings number = 10 or 11, 3 if: meetings number = 8 or 9, 2 if: meetings number = 6 or 7, 1 if: meetings number = 4 or 5, 0 otherwise
BDI(5)	Presence of women	This criterion equals to: 5 if the presence of women is:> 50% 4 if: 40% < % presence of women <50%, 3 if: 30% < % presence of women <40%, 2 if: 20% < % presence of women <30%, 1 if: 10% < % presence of women <20%, 0 otherwise
BDI(6)	Experience average of members	This criterion equals to: 5 if the average experience of board member is greater than 10 years 4 if: 9 < experience average of board member <10 3 if: 8 < experience average of board member <9 2 if: 7 < experience average of board member <8 1 if: 6 < experience average of board member <7 0 otherwise
BDI(7)	Experience majors of board members (Management skills, corporate governance, strategic planning, compensation, risk management, finance, IT, regulation)	This criterion equals to: 5 if the experience of all board members exists in eight majors, 4 if: 4 majors < the experience of all board members <7 majors 3 if: 3 < the experience of all board members <6 2 if: 2 < the experience of all board members <5 1 if: 1 < the experience of all board members <4 0 otherwise
BDI(8)	Training hours in banking industry received	This criterion equals to: 5 if the members of board followed training related to banking sector 0 otherwise
BDI(9)	Chairman independence	This criterion equals to 5 if the chairman is NED 0 otherwise
BDI(10)	Number of positions board members	This criterion equals to 5 if all board members do not hold another position within the same bank 0 otherwise
BDI(11)	Information related to corporate governance	This criterion equals to 5 if the bank published information related corporate governance 0 otherwise
BDI(12)	Information related to corporate governance code	This criterion equals to 5 if the bank defined its own codes related to corporate governance 0 otherwise
BDI(13)	Number of committees (Nomination, Compliance, Internal Audit, Risk Management, Remuneration committees)	This criterion equals to: 5 if there are 5 efficient committees within the bank 4 if there are 4 efficient committees within the bank 3 if there are 3 efficient committees within the bank 2 if there are 2 efficient committees within the bank 1 if there are 1 efficient committee within the bank 0 otherwise
**Total Index Value**		**(**$\sum_{i=1}^{13}BDI.i$**)/13**

### 2.3 Risk management committee indicator (RMCI)

In their corporate governance report, OECD [5] and BASEL [3] stated that risk management committee is an important tool used bankers to measure, control and manage all types of banks risks such as credit, financial, operational and market risks. In this regard, they proposed several recommendations to improve the performance of risk management committee. Numerous scholars and regulators in banking sector [3,20] stated that the risk management committee should be managed by independent and professional Chief Risk Officer who must considers and mitigate all risk types. Others [21,22] have focused on other criteria related to committee experience and meetings.

Based on the literature review, the performance of the relevant committee is based on the criteria presented in Table 2.

**Table 2:** Risk management committee indicator (RMCI).

Symbol	Criterion	Value of each criterion
RMCI(1)	Presence of CRO	This criterion equals to 5 in case of the existence of independent CRO 0 otherwise
RMCI(2)	Separation between CRO and other positions	This criterion equals to 5 if the CRO position does not hold another position 0 otherwise
RMCI(3)	Meetings of committee	This criterion equals to: 5 if the meeting number is 12 and above per year 4 if: meetings number = 10 or 11 per year, 3 if: meetings number = 8 or 9 per year, 2 if: meetings number = 6 or 7 per year, 1 if: meetings number = 4 or 5 per year, 0 otherwise
RMCI(4)	Direct reporting to the board	This criterion equals to 5 if the committee reports to the board members and CEO 0 otherwise
RMCI(5)	Independent members in the committee	This criterion equals to 5 if the % of independent is above 66% 0 otherwise
RMCI(6)	Experience of risk committee	This criterion equals to: 5 if the average experience of board member is greater than 10 years 4 if: 9 < experience average of committee members <10 3 if: 8 < experience average of committee members <9 2 if: 7 < experience average of committee members <8 1 if: 6 < experience average of committee members <7 0 otherwise
RMCI(7)	Size of risk committee	This criterion equals to 5 if the number of members of risk committee is greater than 3 0 otherwise
RMCI(8)	Policies, procedures, and quantitative models of risk management	This criterion equals to 5 if the bank has plans, quantitative simulations, and specific procedures 0 otherwise
RMCI(9)	Diversification	This criterion equals to 5 if the bank has no lending exposure to just one client 0 otherwise
**Total Index Value**		**(**$\sum_{i=1}^{9}RMCI.i$**)/9**

### 2.4 Internal audit committee indicator (IACI)

An independent internal audit committee is a key success factor within banking sector due to its role in controlling and monitoring the operational and financial processes [23]. This committee also must monitor the governance structure and control all types of procedures. The banking financial regulators like BASEL and IMF [[1], [2], [3]] have recommended banks to hire a professional internal audit committee to safeguard the interests of depositors and banks owners. They recommended bankers to make this committee independent from banks CEOs. Numerous scholars [19,21] have provided several recommendations related to experience and size levels to improve the role of this committee within banking sector.

Based on the literature review, the performance of internal audit committee is based on the criteria presented in Table 3.

**Table 3:** Internal audit committee indicator (IACI).

Symbol	Criterion	Value of each criterion
IACI(1)	Presence of professional chief for the committee	This criterion equals to 5 in case of the existence of independent chief for the committee 0 otherwise
IACI(2)	Separation of audit committee and other ranks	This criterion equals to 5 if the committee members do not hold another position 0 otherwise
IACI(3)	Meetings of committee	This criterion equals to: 5 if the meeting number is 12 and above per year 4 if: meetings number = 10 or 11 per year, 3 if: meetings number = 8 or 9 per year, 2 if: meetings number = 6 or 7 per year, 1 if: meetings number = 4 or 5 per year, 0 otherwise
IACI(4)	Direct reporting to the board	This criterion equals to 5 if the committee reports to the board members without passing by CEO 0 otherwise
IACI(5)	Independent members of internal audit committee	This criterion equals to 5 if the % of independent is 100% 0 otherwise
IACI(6)	Experience of internal audit committee	This criterion equals to: 5 if the average experience of board member is greater than 10 years 4 if: 9 < experience average of committee members <10 3 if: 8 < experience average of committee members <9 2 if: 7 < experience average of committee members <8 1 if: 6 < experience average of committee members <7 0 otherwise
IACI(7)	Size of internal audit committee	This criterion equals to 5 if the number of members of internal audit committee is greater than 3 0 otherwise
IACI(8)	Internal member of committee direct report to the committee	This criterion equals to 5 if all auditors report directly to the committee 0 otherwise
**Total Index Value**		**(**$\sum_{i=1}^{8}IACI.i$**)/8**

### 2.5 Remuneration committee indicator (RCI)

In banking sector, the remuneration committee must elaborate the compensation strategy of executives and employees. The committee defines the incentive plans to increase the financial performance of banks and decrease any kind of conflicts between agent and principle [6]. This committee should collaborate with the board of directors to guarantee that the remuneration fulfils with bank strategy and general regulations. This committee has to be professional and independent to eliminate any manipulation by the CEO of banks [14]. Based on the literature review, the performance of remuneration committee index should be based on the criteria presented in Table 4.

**Table 4:** Remuneration committee indicator (RCI).

Symbol	Criterion	Value of each criterion
RCI(1)	Presence of professional chief for the committee	This criterion equals to 5 in case of the existence of independent chief for the committee 0 otherwise
RCI(2)	Separation of remuneration committee and other ranks	This criterion equals to 5 if the committee members do not hold another position 0 otherwise
RCI(3)	Meetings of committee	This criterion equals to: 5 if the meeting number is 12 and above per year 4 if: meetings number = 10 or 11 per year, 3 if: meetings number = 8 or 9 per year, 2 if: meetings number = 6 or 7 per year, 1 if: meetings number = 4 or 5 per year, 0 otherwise
RCI(4)	Direct report to the board	This criterion equals to 5 if the committee reports to the board members without passing by CEO 0 otherwise
RCI(5)	Independent members of compensation committee	This criterion equals to 5 if the % of independent is 66% 0 otherwise
RCI(6)	Experience of remuneration committee	This criterion equals to: 5 if the average experience of board member is greater than 10 years 4 if: 9 < experience average of committee members <10 3 if: 8 < experience average of committee members <9 2 if: 7 < experience average of committee members <8 1 if: 6 < experience average of committee members <7 0 otherwise
RCI(7)	Size of remuneration committee	This criterion equals to 5 if the number of members of remuneration committee is greater than 3 0 otherwise
RCI(8)	Compensation strategy	This criterion equals to 5 if the committee defined a pay-performance structure 0 otherwise
**Total Index Value**		**(**$\sum_{i=1}^{8}RCI.i$**)/8**

### 2.6 Ethics and compliance committee indicator (CECI)

BASEL [3,4] and scholars [24] have recommended banks to empower the role of an ethics committee. The mission of this committee is to define and control the practices of ethical codes and professional policies. The ethics and compliance committee must ensure that bank is respecting the international regulations defined by regulators at the local and international level. The existence of compliance and ethics committee could lead to increase the financial performance banks [25]. They provide several recommendations related to transparency and independency to increase the efficiency of the committee.

Based on the literature review, the performance of compliance and ethics committee is based on the criteria presented in Table 5.

**Table 5:** Ethics and compliance committee indicator (CECI).

Symbol	Criterion	Value of each criterion
CECI(1)	Presence of professional chief for the committee	This criterion equals to 5 in case of the existence of independent chief for the committee 0 otherwise
CECI(2)	Separation between compliance committee and other functions	This criterion equals to 5 if the committee members do not hold another position 0 otherwise
CECI(3)	Meetings of committee	This criterion equals to: 5 if the meeting number is 12 and above per year 4 if: meetings number = 10 or 11 per year, 3 if: meetings number = 8 or 9 per year, 2 if: meetings number = 6 or 7 per year, 1 if: meetings number = 4 or 5 per year, 0 otherwise
CECI(4)	Direct report to the board	This criterion equals to 5 if the committee reports to the board members without passing by CEO 0 otherwise
CECI(5)	Independent members of compliance and ethics committee	This criterion equals to 5 if the % of independent is 66% 0 otherwise
CECI(6)	Experience of compliance and ethics committee	This criterion equals to: 5 if the average experience of board member is greater than 10 years 4 if: 9 < experience average of committee members <10 3 if: 8 < experience average of committee members <9 2 if: 7 < experience average of committee members <8 1 if: 6 < experience average of committee members <7 0 otherwise
CECI(7)	Size of compliance and ethics committee	This criterion equals to 5 if the number of members of compliance and ethics committee is greater than 3 0 otherwise
CECI(8)	Compliance and ethics codes	This criterion equals to 5 if there are a well-defined ethics and compliance codes 0 otherwise
**Total Index Value**		**(**$\sum_{i=1}^{8}CECI.i$**)/8**

### 2.7 Nominating committee indicator (NCI)

The nominating (and governance) committee plays an important role within banking sector because it supports the board of directors in nominating and selecting seniors and executives. This committee must verify that the selected members on board of directors and senior positions fill the professional requirements. In the same line, BASEL [3,4] have recommended banks to hire a professional and efficient nomination committee that is able to monitor the performance of board members and CEOs. For some scholars [21,25,26], the nomination committee must fill several the requirements of independency and experience to reach its mission. Based on the literature review, the basic requirements of efficient nomination committee index are presented in Table 6.

**Table 6:** Nominating (governance) committee indicator (NCI).

Symbol	Criterion	Value of each criterion
NCI(1)	Presence of professional chief for the committee	This criterion equals to 5 in case of the existence of independent chief for the committee 0 otherwise
NCI(2)	Separation between nomination committee and other functions	This criterion equals to 5 if the committee members do not hold another position in banking sector 0 otherwise
NCI(3)	Meetings of committee	This criterion equals to: 5 if the meeting number is 12 and above per year 4 if: meetings number = 10 or 11 per year, 3 if: meetings number = 8 or 9 per year, 2 if: meetings number = 6 or 7 per year, 1 if: meetings number = 4 or 5 per year, 0 otherwise
NCI(4)	Direct report to board members	This criterion equals to 5 if the committee reports to the board members 0 otherwise
NCI(5)	Independent members of nomination committee	This criterion equals to 5 if the % of independent is 66% 0 otherwise
NCI(6)	Experience level of nomination committee	This criterion equals to: 5 if the average experience of board member is greater than 10 years 4 if: 9 < experience average of committee members <10 3 if: 8 < experience average of committee members <9 2 if: 7 < experience average of committee members <8 1 if: 6 < experience average of committee members <7 0 otherwise
NCI(7)	Size of nomination committee	This criterion equals to 5 if the number of members of nomination committee is greater than 3 0 otherwise
NCI(8)	Committee succession plan and defined system	This criterion equals to 5 if the committee have a succession plan and evaluation system for HR 0 otherwise
**Total Index Value**		**(**$\sum_{i=1}^{8}NCI.i$**)/8**

### 2.8 Disclosing and transparency level indicator (DTLI)

The last corporate governance index is related to disclosure and transparency. This dimension of governance mechanism is executed by bankers but provided for both internal and external stakeholders. The main objective of being transparent is to help all banks' stakeholders to control CEOs and other relevant executives. Furthermore, it provides a clear vision about the financial and non-financial situation of the bank. The OECD [5] and BASEL [3,4] recommended banks to be transparent and disclose the information to shareholders, debtholders, regulators and depositors. For Tarchouna et al. [24], banks must provide their information related to strategy, ownership, financial data, boards, remuneration and risks via their annual reports. Based on literature review, the disclosure of banks must include the elements presented in Table 7.

**Table 7:** Disclosing and transparency level indicator (DTL).

Symbol	Criterion	Value of each criterion
DTL(1)	Existing of payment information	This criterion equals to 5 if all the payment information and data of executives are published in the annual report 0 otherwise
DTL(2)	Existing of financial information	This criterion equals to 5 if the financial information and data are published in the annual report 0 otherwise
DTL(3)	Existing of ownership information	This criterion equals to 5 if the ownership structure information and data are published in the annual report 0 otherwise
DTL(4)	Existing of risk management information	This criterion equals to 5 if the risk management information and data are published in the annual report 0 otherwise
DTL(5)	Existing of non-compliance information	This criterion equals to 5 if the non-compliance information and data are published in the annual report 0 otherwise
DTL(6)	Existing of board of directors' information	This criterion equals to 5 if the board of directors' characteristics are published in the annual report 0 otherwise
DTL(7)	Existing of executives committees' information	This criterion equals to 5 if the executives committees' information and data are published in the annual report 0 otherwise
DTL(8)	Existing of accounting and financial information	This criterion equals to 5 if the accounting and financial system information and data are published in the annual report 0 otherwise
DTL(9)	Existing of strategic information	This criterion equals to 5 if the strategic information and data are published in the annual report 0 otherwise
DTL(10)	Existing of dividends' information	This criterion equals to 5 if the dividends' information and data are published in the annual report 0 otherwise
DTL(11)	Existing of stocks' transaction information	This criterion equals to 5 if the stocks' transaction information and data are published in the annual report 0 otherwise
DTL(12)	Existing of third-party transaction information	This criterion equals to 5 if the third party transaction information and data are published in the annual report 0 otherwise
DTL(13)	Existing of stocks owned by executives' information	This criterion equals to 5 if the stocks owned by executives' information and data are published in the annual report 0 otherwise
DTL(14)	Existing of CEO, CRO, CFO, and other executives' information	This criterion equals to 5 if the executives' information and data are published in the annual report 0 otherwise
DTL(15)	Existing of managerial’ information	This criterion equals to 5 if the managerial’ (such as managers names) information and data are published in the annual report 0 otherwise
DTL(16)	Existing of whistle blower policy information	This criterion equals to 5 if the whistle blower policy information and data are published in the annual report 0 otherwise
**Total Index Value**		**(**$\sum_{i=1}^{16}DTL.i$**)/16**

## 3 Formulation of the governance index of banking sector

**Fig. 1:**
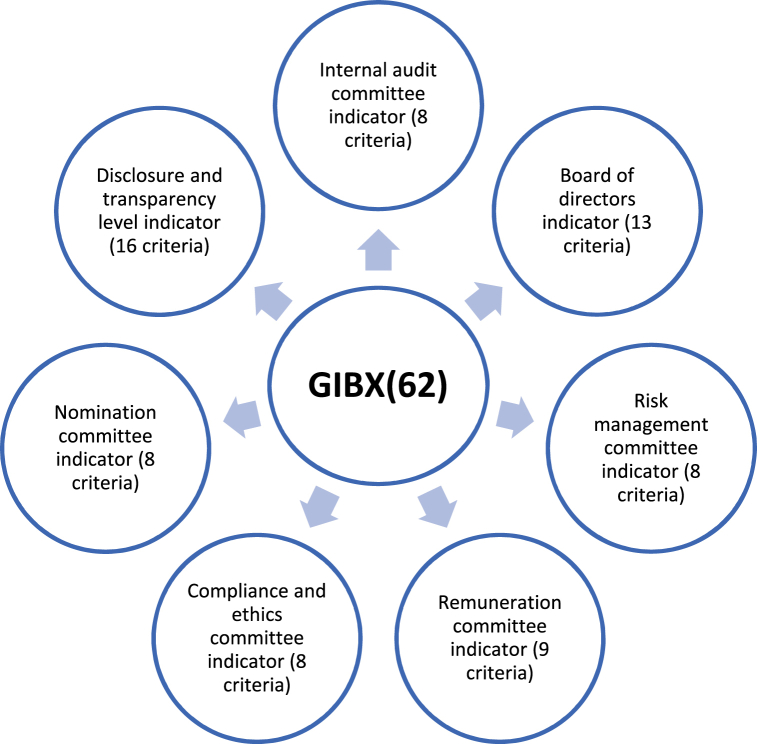
GIB.X(62).

After presenting the different important components of efficient corporate governance in banking sector. This section presents the GIBX(62) (Governance Index of Banking sector), formulated based on 62 internal indicators and 7 constructs. The GIB.X62 can be generalized and used as a tool to monitor the governance structure and performance of banks by depositors, investors, regulators and the shareholders. This index can provide two set of information based on its two different indicators: the first set of information is related to the global governance mechanisms in banking sector and the second set of information is related to the different internal governance mechanisms namely bank risks, remuneration, disclosure, transparency, ethics, board of directors and internal audit. Fig. 1 summarizes the two sets of information GIB.X62 provides.

### 3.1 Annual banking governance index value: (GIB.X(62))_y1_

Based on the data presented in Table 1, Table 2, Table 3, Table 4, Table 5, Table 6, Table 7, the following formula could be stated:

The yearly governance performance indicators for the banking sector is measured by the following formula:

(1) $${\left(\text{GIB}.X62\right)}_{y1}=\frac{\text{BDI}.x13+\text{RMCI}.x9+\text{IACI}.x8+\text{RCI}.x8+\text{CECI}.x8+\text{NCI}.x8+\text{DTL}.x16}{7}$$

(2) $$\Rightarrow {\left(\text{GIB}.X62\right)}_{y1}=\frac{\left(\sum_{i=1}^{13}BDI.i\right)}{13}+\frac{\left(\sum_{i=1}^{9}RMCI.i\right)}{9}+\frac{\left(\sum_{i=1}^{8}IACI.i\right)}{8}+\frac{\left(\sum_{i=1}^{8}RCI.i\right)}{8}+\frac{\left(\sum_{i=1}^{8}CECI.i\right)}{8}+\frac{\left(\sum_{i=1}^{8}NCI.i\right)}{8}+\frac{\left(\sum_{i=1}^{16}DTL.i\right)}{16}$$

**Fig. 2:**
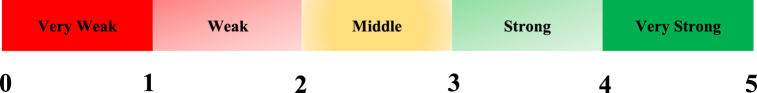
Different levels of performance of GIB.X(62)_y1_.

The value of GIB.X(62)_y1_ must be between 0 and 5. Fig. 2 highlights the different levels of performance of GIB.X(62). The corporate governance index is very strong if the value of index is between 4 and 5, strong if the value of index is between 3 and 4, middle if the value of index is between 2 and 3, weak if the value of index is between 1 and 2 and very weak if the value of index is between 0 and 1.

### 3.2 Application of GIB.X(62)_y1:_ Analytical study on 7 banks

This section consists of testing the defined GIB.X(62)_y1_ index on 7 different banks.

An email has been sent to 38 banks from 19 countries during the first quarter in 2021. The selection of this sample was based on the available data in Orbis Bank focus database. Only 7 banks from 7 countries accepted to complete the application of GIB.X(62)_y1_ index.

The first bank was from US market, three other banks were from European market and the last three banks were from three developing countries in MENA region. The data related to corporate governance were extracted from the website of each bank, published annual report and the complementary data have been received after a direct email that has been sent to each bank. The sample of the study is presented in Table 8.

**Table 8:** Sample of the case study.

Region		Country	Number of the banks
US		US	1
Europe		France	1
		Spain	1
		Italy	1
MENA region	Africa	Egypt	1
	Asia	Lebanon	1
		Jordan	1
Total →			7

**Fig. 3:**
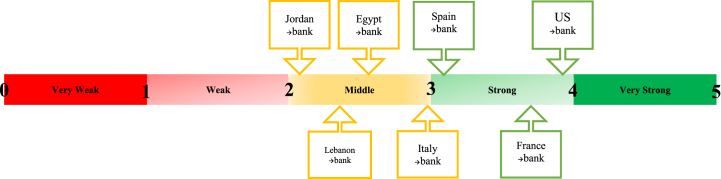
Classification of banks based on GIBX(62)index.

The results in Table 9 presents the GIB.X(62)_y1_ and the 7 internal governance indicators. The results show the indicator differences between the 7 banks. It is very clear from the results of Table 9 that the indexing value of corporate governance in the three selected banks in MENA region is on the average (between 2.11 and 2.51). Thus, a reinforcement of governance mechanisms is required in three banks in MENA region, as can be seen in Fig. 3. The indexing value is greater in Europe and US.

**Table 9:** Governance indicators values of 7 different banks.

Region→		US	Europe			Asia		Africa
Country→		US	France	Italy	Spain	Lebanon	Jordan	Egypt
Name of the indicator	Symbol	Mean	Mean	Mean	Mean	Mean	Mean	Mean
Board of director performance indicator	BDI.x13	4.1	4	3.6	3.3	2.6	2.3	2.1
Risk management performance indicator	RMCI.x9	4.1	3.2	3.5	2.6	3.3	2.3	2.7
Internal audit performance indicator	IACI.x8	3.8	3.2	3	3.2	2.5	2.2	3.1
Compensation performance indicator	RCI.x8	4.2	4.4	2.5	3.6	2	2.1	2.5
Compliance and ethics performance indicator	CECI.x8	3	3.3	2.7	3.4	2	1.5	2.3
Nomination performance indicator	NCI.x8	3.3	3.7	2.4	2.4	2.4	2.1	2.4
Disclosure and transparency performance indicator	DTL.x16	4.2	4.1	3.2	3.3	2.1	2.4	2.2
Banking Global Index	**(**GIB.X62)_y1_	3.81	3.7	2.98	3.11	2.41	2.11	2.51
Results →		Strong	Strong	Middle	Strong	Middle	Middle	Middle

**Fig. 4:**
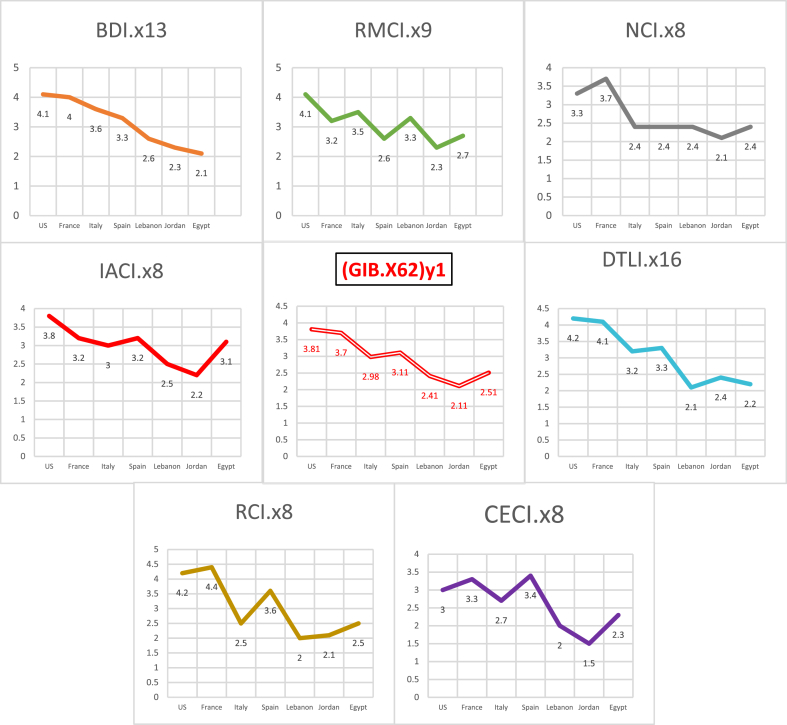
Internal performance indicators and GIB.X(62)index.

The results in Fig. 4 present the different internal performance indicators of the corporate governance mechanisms in the banking sector. The highest the value of indicator, the better the corporate governance mechanism is. All the internal indicators of corporate governance tools (BDI- board of directors, IACI- internal audit, RCI- compensation control, RMCI- risk management control, CECI- compliance and ethics, NCI- nomination, DLTI- transparency and disclosure, and GIB.X(62)-global index) show that the US banks and the European banks except Italy have strong indicators (3 and above). Their corporate governance mechanisms are efficient. Their owners and debtholders can monitor any expropriation behavior of CEO and internal owner.

In addition, the results reveal that the internal performance indicators are weak in MENA region, mainly in the three selected banks from Lebanon, Jordan and Egypt (below 3). Thus, in MENA region banks have high expropriation risks since the efficiency level of their corporate governance mechanisms is relatively low. The in-depth analysis of MENA region shows that the global index of corporate governance in Egypt (Africa) is higher than the average values in Asia, namely Lebanon and Jordan.

The analysis of the results presented above could be correlated with level of governments and financial regulators power and authority. In case of low legal protection, the management of banks might misuse their position to control the corporate governance tools and thus, to only think of their private benefits. In case of low regulation level, the management in banks is not capable manipulate the corporate governance structure and extract private benefits since they are under the control of governments and financial regulators.

In total, the proposed corporate governance indicators offer to bankers an efficient tool to control any expropriation behavior and mitigate all types of financial and operational risks. The indicators provide bankers to match their corporate governance practices in the same country and even in different countries.

## 4 Discussion

In the literature there are many corporate governance studies that revealed the direct and indirect impact of corporate governance tools used by banks on the financial risk and performance. Numerous studies have shown that the use of effective corporate governance practices can significantly reduce financial risk and improve financial performance [11]. To give examples, independent boards of directors, transparency in financial reporting and effective internal controls have been shown to be positively related to financial stability. On the other hand, poor governance practices, such as weak internal controls and a lack of transparency, have been associated with increased financial risk and decreased financial performance [27].

The literature has also explored the indirect impact of corporate governance on financial risk and performance. For example, effective corporate governance practices can enhance the reputation of a bank and increase public trust, which can in turn lead to increased customer loyalty and improved financial performance [28].

The common problem in the previous studies is the absence of corporate governance measures and indexes that can be generalized and used worldwide in banking sector. For example, the BASEL committee recommended the employment of corporate governance improvements to protect banking sector against any international crisis without mentioning any appropriate model that can be easily implemented.

This research succeeded to propose and test a new corporate governance model able to be implemented by banks regardless their regulation and monetary system. The proposed index has several advantages like its capacity to be easily used by banks. Furthermore, this index could be used by bankers, owners and even depositors to evaluate the transparency, managerial efficiency, and governance mechanisms within banks.

The proposed index also could overcome the disadvantages of existing indexes since it covers all the managerial dimensions that must controlled by bankers and other relevant stakeholders.

Having discussed the advantages of the proposed new index, it is important to acknowledge its limitations, which should be addressed in future research. On the one hand, the scope of this study is limited to the banks of seven different countries. Further research should replicate the study in a wider range of banks in multiple countries to validate the results and compare them across different banking systems. Additionally, we have limited ourselves to seven performance indicators. We believe that these seven indicators cover performance, but would leave it to future research to add or remove indicators. Lastly, and related to the last point, it was the intention of the authors, we tried to come up with an index that is simple to use by banks. Adding some complexity to the index in form of indicators might increase the validity of results.

## 5 Conclusion

This main objective of this paper is to define a new index to measure the corporate governance structure in the banking sector. This novel index was built based on 7 internal corporate governance constructs and 62 criteria related to: (1) board of directors, (2) internal audit, (3) compensation control, (4) risk management control, (5) compliance and ethics, (6) nomination, and (7) transparency and disclosure. All the constructs and criteria were based on the recommendations of scholars, experts, international financial regulators and institutions like BASEL and OECD.

In addition, the GIBX(62)index was tested on 7 banks from 7 different countries. The results of this test on 7 banks revealed that the application of this new index is very simple and easy to be practiced by banks in order to measure the efficiency of the governance mechanisms worldwide. The results also showed that the highest level of corporate governance efficiency exists in US market. The corporate governance efficiency is high in Spain and France and finally, the level of governance efficiency become middle level in Italy, Egypt, Lebanon, and Jordan.

This research has several theoretical and managerial implications. First, this paper adds novel insights to the literature by creating a new corporate governance index that could be applied by banks worldwide. Second, the index can help the directors to detect and improve the shortcomings of internal governance mechanisms in banking sector. Third, the GIB.X(62) also can be used by regulators (inter)nationally.

Despite the theoretical and managerial contributions, this paper has several limitations that could be addressed in future research works. First, the proposed index was built based on internal tools of corporate governance without considering the external dimensions. Second, the corporate governance index was tested on limited number of banks. Third, the proposed index was constructed based on limited number of constructs and criteria. Thus, a future research works could be conducted on larger number of banks by considering different periods and external corporate governance dimensions.

## Author contribution statement

Hani El-Chaarani; Zouhour El-Abiad: Conceived and designed the experiments; Performed the experiments; Analyzed and interpreted the data; Contributed reagents, materials, analysis tools or data; Wrote the paper.

Udo Braendle: Conceived and designed the experiments; Performed the experiments; Contributed reagents, materials, analysis tools or data; Wrote the paper.

## Funding statement

This research did not receive any specific grant from funding agencies in the public, commercial, or not-for-profit sectors.

## Data availability statement

Data included in article/supplementary material/referenced in article.

### Declaration of interest’s statement

The authors declare no conflict of interest.
